# Left Ventricular Longitudinal Strain Is Associated with Ascending Aortic Diameters in Healthy Adults—A Detailed Single-Center Retrospective Analysis from the Three-Dimensional Speckle-Tracking Echocardiographic MAGYAR-Healthy Study

**DOI:** 10.3390/medicina62081522

**Published:** 2026-08-07

**Authors:** Attila Nemes, Nóra Ambrus, Csaba Lengyel

**Affiliations:** Department of Medicine, Albert Szent-Györgyi Medical School, University of Szeged, Semmelweis Street 8, P.O. Box 427, H-6725 Szeged, Hungary; ambrusnora@gmail.com (N.A.); lengyel.csaba@med.u-szeged.hu (C.L.)

**Keywords:** speckle-tracking, echocardiography, aorta, healthy, association, left ventricular, strain

## Abstract

*Background and Objectives*: A more precise understanding of ventriculo–arterial coupling is essential, particularly regarding quantitative and qualitative associations can be demonstrated between the aorta and the left ventricle (LV), which is considered the engine of the central circulation. The aim of the present study was to investigate the associations between three-dimensional speckle-tracking echocardiography (3DSTE) derived unidimensional LV strains and end-systolic and end-diastolic ascending aortic diameters measured by M-mode echocardiography (MME). Additionally, the outcomes were analyzed after stratifying the parameters into below-average, average, and above-average. *Materials and Methods*: The present retrospective cohort study consisted of 162 healthy adult volunteers enrolled between 2011 and 2017, of which 93 participants (mean age: 30.6 ± 10.0 years; 54 males) were eligible for simultaneous 3DSTE-derived LV strain assessment and MME-based aortic dimension measurement. In all individuals, MME, two-dimensional Doppler echocardiography and 3DSTE were performed. *Results*: An increase in end-diastolic ascending aortic diameter (AAD) was associated with an increase in end-systolic AAD, and vice versa. At the maximum end-diastolic AAD, the basal, global, and mean segmental LV longitudinal strain (LS) values reached their lowest. When end-systolic AAD reached its maximum, both global and mean segmental LV-LS dropped to their minimum. The highest end-diastolic and end-systolic AADs were detectable in the presence of the lowest global LV-LS. With larger end-diastolic and end-systolic AADs, a trend toward higher LV radial strain was present. *Conclusions*: Even in the absence of cardiovascular pathology, a finely tuned interaction can be observed between regional LV deformation, especially with LV-LS and the size of the ascending aorta in healthy individuals.

## 1. Introduction

Recent technological advances in medical imaging have enabled detailed analyses in large patient cohorts, allowing the evaluation of specific, even physiological, processes. A more precise and detailed understanding of ventriculo–arterial coupling is needed, particularly regarding the quantitative and qualitative associations between the aorta and the left ventricle (LV) [1,2,3]. Physiologically, the ascending aorta acts as a critical modulator of LV afterload; changes in aortic compliance and dimensions directly influence myocardial wall stress and retrograde wave reflections, which in turn modulate regional LV myocardial contraction [1,2,3]. However, the exact mechanical interplay between non-invasively assessed aortic geometry and multidirectional subclinical LV deformation remains poorly understood. State-of-the-art three-dimensional (3D) speckle-tracking echocardiography (3DSTE) enables detailed deformation analysis on the LV using regional strains derived from virtual, accurate 3D casts of the LV [4,5,6,7,8,9,10,11,12]. During the cardiac cycle, the ascending aorta undergoes cyclic systolic and diastolic diameter changes, which can be simultaneously measured using easily performable M-mode echocardiography (MME) [13]. While our research group has previously investigated correlations between LV functional properties and aortic elastic properties [14,15], a critical knowledge gap persists regarding how localized aortic cyclic dynamics relate to specific LV strains representing LV deformation. This study expands upon our prior findings by uniquely focusing on the direct correlation between MME-derived ascending aortic diameter changes and localized 3DSTE-derived unidimensional strain parameters within the same cohort. The aim of the present study was to investigate the associations between 3DSTE-derived unidimensional LV strain parameters representing 3D LV contraction pattern and ascending aortic diameter changes measured by MME. In addition, we compared these parameters after categorizing them as below average, average, or above average to explore potential non-linear mathematical relationships dependent on the cohort’s distribution trends. We present this article in accordance with the STROBE reporting checklist.

## 2. Subjects and Methods

The present retrospective cohort study consisted of 162 healthy adult volunteers enrolled between 2011 and 2017, of which 93 participants (mean age: 30.6 ± 10.0 years; 54 males) were eligible for simultaneous 3DSTE-derived LV strain assessment and MME-based aortic dimension measurement. All participants demonstrated normal clinical profiles, as verified by unremarkable physical examinations, standard 12-lead electrocardiograms (ECGs), routine laboratory screens, MME and conventional two-dimensional (2D) Doppler echocardiography. Individuals were excluded from the study if they presented with obesity (defined as a body mass index >30 kg/m^2^), pregnancy, current smoking habits, regular medication usage, or regular athletic training. None of the enrolled subjects suffered from acute or chronic medical conditions that might have skewed the study outcomes. This research was conducted under the framework of the ‘**M**otion **A**nalysis of the heart and **G**reat vessels b**Y** three-dimension**A**l speckle-t**R**acking echocardiography in **Healthy** subjects’ (**MAGYAR-Healthy**) **Study**, an ongoing project investigating normal cardiac mechanics using 3DSTE-derived parameters (the word ’Magyar’ translates to ’Hungarian’). The study adhered to the principles of the Declaration of Helsinki (revised in 2013) and was approved by the Institutional and Regional Human Biomedical Research Committee at the University of Szeged, Hungary (protocol number: 71/2011, original approval 23 May 2011, latest approval on 17 March 2025). All participants provided written informed consent prior to enrollment.

**Figure 1:**
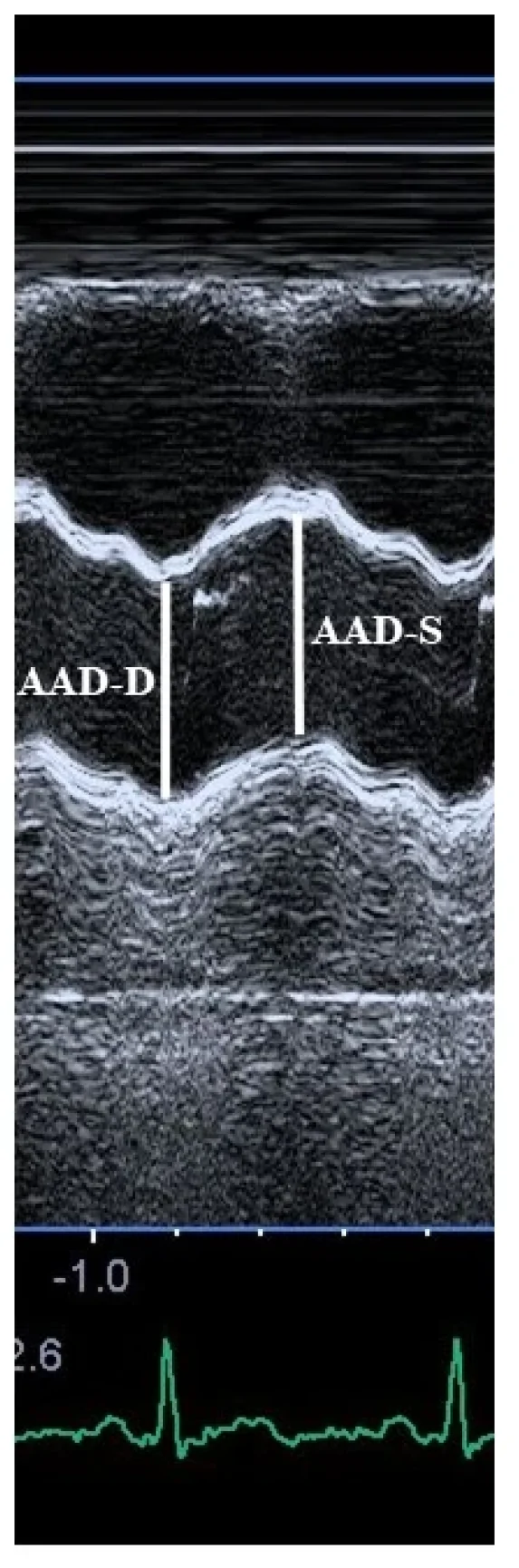
Measurements of end-systolic (AAD-S) and end-diastolic (AAD-D) diameters of the ascending aorta are shown on the M-mode tracing obtained 3 cm above the aortic valve.

**M-mode and Two-dimensional Doppler Echocardiography.** Standard 2D echocardiographic evaluations were performed using a Toshiba Artida^®^ ultrasound system (Toshiba Medical Systems, Tokyo, Japan) paired with a 1–5 MHz PST-30BT broadband phased-array transducer. This equipment was used to measure left atrial dimensions and left ventricular (LV) volumes, while the modified Simpson’s technique was applied to calculate the LV ejection fraction (EF). Valvular pathologies, including hemodynamically significant stenosis or regurgitation, were excluded via Doppler echocardiography. Additionally, transmitral diastolic flow profiles were recorded to establish early (E) and late (A) diastolic filling velocities, along with the corresponding E/A ratio [16]. End-diastolic and end-systolic ascending aortic diameters (AAD-D and AAD-S, respectively) were measured by MME 3 cm above the aortic valve from a parasternal long-axis view. AAD-D was measured at the peak of QRS complex, whereas AAD-S was measured at the point of maximal aortic anterior motion at the end of T wave [13] (Figure 1).

**Figure 2:**
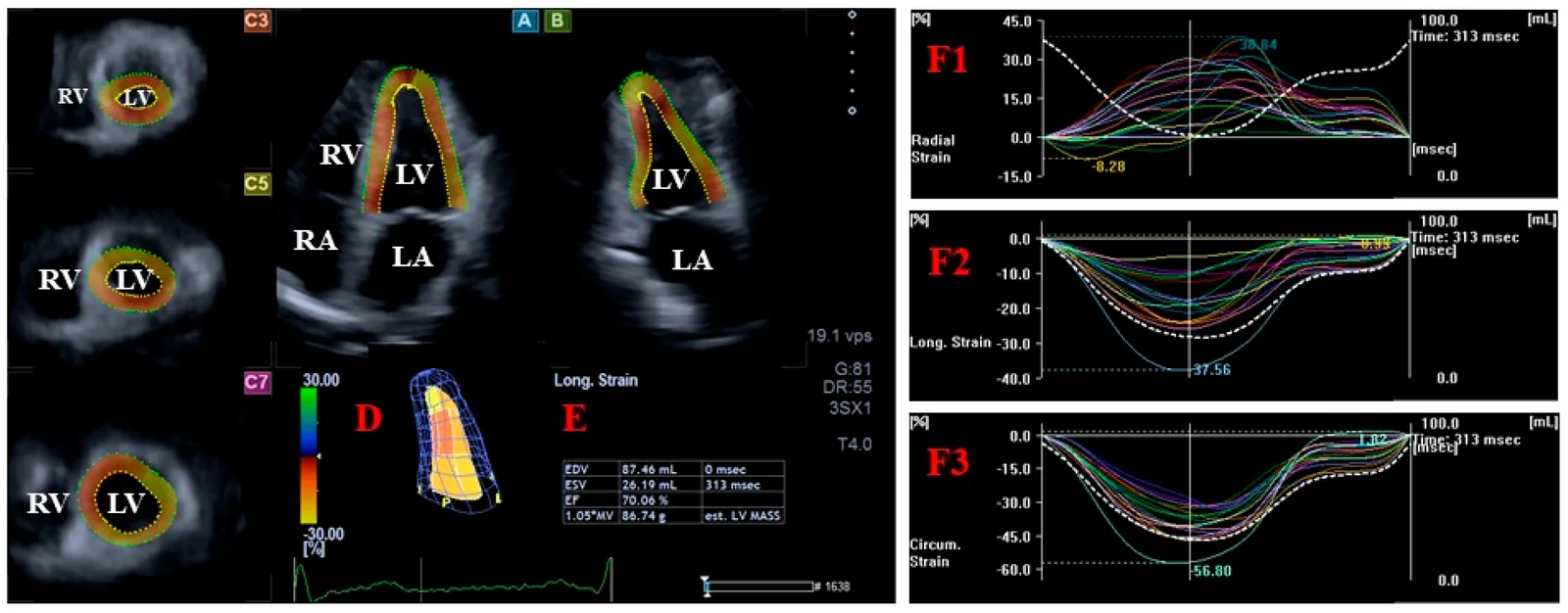
Assessment of left ventricular (LV) strain parameters using three-dimensional (3D) speckle-tracking echocardiography. The panel displays the apical four-chamber long-axis view (**A**), the apical two-chamber long-axis view (**B**), and the basal (**C3**), midventricular (**C5**), and apical (**C7**) short-axis views, all generated via automated extraction from the captured 3D echocardiographic dataset. Panel (**D**) illustrates the reconstructed 3D geometric cast of the LV, and panel (**E**) provides the calculated LV volumes along with the LV ejection fraction. The accompanying graphs present the global (white line) and segmental (colored lines) time–LV strain curves for radial, longitudinal, circumferential deformation (**F1**–**F3**). **Abbreviations:** LV, left ventricle; LA, left atrium; RV, right ventricle; RA, right atrium.

**Three-Dimensional Speckle-Tracking Echocardiography.** The 3DSTE procedure followed a two-step workflow [7,8,9,10,11,12]. First, data were collected using a Toshiba Artida^®^ system paired with a 3D-enabled PST-25SX matrix-array transducer (Toshiba Medical Systems, Tokyo, Japan). After fine-tuning the gain and depth settings, full-volume 3D datasets of the LV were acquired from the apical window. To guarantee optimal image quality, acquisitions were completed during a single breath-hold across six consecutive cardiac cycles, ensuring stable ECG RR-intervals. The system software then automatically stitched these separate subvolumes together. Second, data underwent post-processing via proprietary 3D Wall Motion Tracking software (version 2.7, UltraExtend, Toshiba Medical Systems, Tokyo, Japan). This analysis integrated apical two- and four-chamber long-axis views with three LV short-axis views taken at the basal, midventricular, and apical planes. After manual image adjustments, the operator positioned reference markers along the LV apical endocardial border and the edges of the LV-mitral annulus. Following a sequential analysis, the software automatically generated a 3D digital reconstruction of the LV endocardial surface. This multidimensional model allowed for the measurement of three separate unidimensional LV strain parameters (Figure 2) [6]:

- Radial strain (RS): reflecting the thickening and thinning dynamics of the LV wall.
- Longitudinal strain (LS): reflecting the shortening and lengthening along the long axis.
- Circumferential strain (CS): reflecting the narrowing and widening of the LV perimeter.

Segmental wall motion was analyzed using the standard 16-segment LV model, which also provided mean segmental and global strain values. Regional LV strains were calculated from respective segmental strains. Overall image quality was assessed on a 5-point Likert scale (ranging from 1 [poor] to 5 [excellent]), with a score of 3 or higher required for inclusion in the final analysis.

**Statistical analysis**. Continuous variables are expressed as mean ± standard deviation (SD), while categorical variables are presented as frequencies and percentages. Normality of data distribution was evaluated using the Shapiro–Wilk test, and the homogeneity of variances was verified via Levene’s test. For comparing two independent groups, the independent-samples t-test was applied to normally distributed data, whereas the Mann–Whitney–Wilcoxon test was used for non-parametric datasets. Multiple-group comparisons were performed using one-way analysis of variance (ANOVA) followed by the Bonferroni post hoc correction, where applicable. For correlations, Pearson’s correlation coefficients were calculated. Multivariable regression analysis has also been performed. A post hoc power analysis was conducted to assess the robustness of the observed differences. Based on the current sample size, the analysis yielded a large effect size (Cohen’s d = 0.8) and a statistical power of 0.68 at an alpha level of 0.05. Intra- and interobserver agreements were tested by interclass correlation coefficients (ICCs). A *p*-value of less than 0.05 was defined as the threshold for statistical significance. All statistical computations were executed using the SPSS software pack (IBM SPSS Statistics for Windows, Version 29.0, IBM Corp., Armonk, NY, USA).

## 3. Results

**Clinical data.** Clinical characteristics and 2D Doppler echocardiographic measurements are presented in Table 1. None of the participants had valvular regurgitation greater than or equal to grade 1, or clinically significant stenosis on any cardiac valve.

**Classification of subjects.** The healthy adult cohort was stratified according to the mean ± SD values of specific 3DSTE and MME parameters. The baseline reference values for this categorization were:

- global LV-RS: 25.5 ± 9.8%;
- global LV-CS: −28.5 ± 5.0%;
- global LV-LS: −16.3 ± 2.6%;
- AAD-D: 29.2 ± 4.1 mm;
- AAD-S: 32.0 ± 4.3 mm.

Based on these mean ± SD intervals, the following specific cut-off values were applied for subject classification:

- global LV-RS: 15.7% and 35.3%;
- global LV-CS: −23.5% and −33.5%;
- global LV-LS: −13.7% and −18.9%;
- AAD-D: 25.1 mm and 33.3 mm;
- AAD-S: 27.7 mm and 36.3 mm.

**Parameters in AAD-D subgroups.** Increasing AAD-D was associated with increasing AAD-S, whereas no association was found with LV-CS parameters. In participants with the highest AAD-D, the basal, global, and mean segmental LV-LS values reached their lowest. Higher LV-RS values tended to be observed with increasing AAD-D (Table 2).

**Parameters in AAD-S subgroups.** Similarly to AAD-D subgroups, the increase in AAD-S was associated with an increase in AAD-D and no association was found regarding the LV-CS parameters. When AAD-S were highest, both global and mean segmental LV-LS were lowest. LV-RS tended to increase with increasing AAD-S (Table 3).

**Parameters in global LV-RS subgroups.** No association was observed between AAD-D and AAD-S, and global LV-RS. Higher global LV-RS was associated with increased regional LV-RS values, as well as higher basal, midventricular, global, and mean segmental LV-CS values. However, no significant relationship was found between LV-RS and LV-LS (Table 4).

**Parameters in global LV-CS subgroups.** Similarly to LV-RS, no association was observed between AAD-D and AAD-S, and global LV-CS. Participants with the highest global LV-CS values also exhibited the highest regional, global, and mean segmental LV-RS with the highest midventricular, global, and mean segmental LV-LS (Table 5).

**Parameters in global LV-LS subgroups.** The highest AAD-D and AAD-S were observed in the presence of the lowest global LV-LS. No association was found between the global LV-LS and all LV-RS parameters. The highest apical LV-CS was observed at the maximum global LV-LS. Increasing global LV-LS was accompanied by corresponding increases in all regional LV-LS values (Table 6).

**Correlations and multiple variable analysis.** AAD-D showed mild correlation with global LV-LS (r = 0.215, *p* = 0.039), but not correlated with global LV-RS (r = 0.094, *p* = 0.372) and global LV-CS (r = −0.011, *p* = 0.917). AAD-S did not correlate with global LV-LS (r = 0.174, *p* = 0.095), global LV-RS (r = 0.046, *p* = 0.660) and global LV-CS (r = 0.005, *p* = 0.965). Multivariable adjustment for key covariates—specifically age (adjusted Odds Ratio [aOR] = 1.05, 95% CI: 0.96–1.12, *p* = 0.43), sex (aOR = 1.03, 95% CI: 0.97–1.10, *p* = 0.47), weight (aOR = 1.06, 95% CI: 0.93–1.16, *p* = 0.48), height (aOR = 1.05, 95% CI: 0.94–1.13, *p* = 0.43), derived body mass index (aOR = 1.04, 95% CI: 0.95–1.13, *p* = 0.43), systolic blood pressure (aOR = 1.09, 95% CI: 0.93–1.18, *p* = 0.51) and diastolic blood pressure (aOR = 1.08, 95% CI: 0.96–1.12, *p* = 0.50), and heart rate (aOR = 1.10, 95% CI: 0.92–1.18, *p* = 0.52)—yielded consistent results. Consequently, the absence of an association cannot be attributed to residual confounding from these parameters.

**Reproducibility of 3DSTE-derived global LV strains.** Intraobserver ICCs for global LV-RS, global LV-CS, and global LV-LS were 0.84, 0.84, and 0.82, respectively (*p* < 0.05 for all). Similarly, intraobserver ICCs for the apical, midventricular, and basal regions were 0.82, 0.83, 0.83; 0.81, 0.82, 0.82; and 0.82, 0.83, 0.83 for LV-RS, LV-CS, and LV-LS, respectively. Interobserver ICCs for the corresponding global parameters were 0.83, 0.79, and 0.78 (*p* < 0.05 for all). Regarding regional interobserver reproducibility, the values were 0.81, 0.79, and 0.79 for the apical; 0.80, 0.78, and 0.78 for the midventricular; and 0.79, 0.79, and 0.78 for the basal segments (for LV-RS, LV-CS, and LV-LS, respectively).

## 4. Discussion

A close interaction exists between the LV and the arterial system, predominantly the aorta, a relationship referred to as ventriculo–arterial coupling. This interaction reflects a dynamic balance between the pumping function of the LV and the resistance of the arterial system. For example, if the afterload is increased, the LV must generate greater work to maintain the cardiac output [1,2,3,17]. In healthy circumstances, the walls of the LV contract in systole, which can be quantitatively characterized by strains in the three directions of space. While the LV shortens longitudinally (represented by a negative LV-LS) and narrows circumferentially (represented by a negative LV-CS), it thickens radially (represented by a positive LV-RS) [6,12]. Consequently, the LV empties by the end of systole, and blood is ejected into the ascending aorta [1]. Because the aorta is an elastic organ, its diameter is at its widest at end-systole. During diastole, the reverse process occurs, while the aortic diameter is at its smallest at end-diastole [1,18]. 3DSTE enables simultaneous assessment of the aforementioned LV strains both globally and segmentally; the method is validated, and its normal values have been established [4,5,7,8,9,10,11,12]. For measuring changes in aortic dimensions, MME has long been an established and widely used technique [13].

Based on the present findings, the following observations and findings can be drawn. First, 3DSTE enables simultaneous determination of all global and regional LV strains measured in the three directions of space, in agreement with literature data [4,5,12]. This finding suggests an opportunity for the simultaneous examination of the complete LV contraction pattern. Second, it was confirmed that aortic dimensions can be measured simultaneously using MME, enabling physiological studies such as the present investigation [13]. Third, as expected, an increase in AAD-D was associated with an increase in AAD-S. However, the increase in different global LV strains was accompanied by distinct patterns of changes in other strains, as previously confirmed in a publication published within the framework of the MAGYAR-Healthy Study [19]. Fourth, the most important finding of the present study is that reduced basal, global, and mean segmental LV-LS can be detected in patients with the largest aortic diameters, regardless of the phase of the cardiac cycle in which it was measured. Conversely, the largest AAD-D and AAD-S were observed in the presence of the lowest global LV-LS. Moreover, this was associated with a non-significant trend toward higher LV-RS. These results may potentially indicate that aortic size is associated with LV strains: in the case of a dilated aorta, lower LV-LS and a non-significant but tendentiously higher LV-RS can be observed, even in healthy subjects having normal aortic dimensions, which may indicate a specific contraction pattern. These findings are particularly interesting because they can be detected even in healthy individuals. Tension and dilation of the aortic wall (e.g., during hypertension or early aortic remodeling) increase LV afterload [20]. Since subendocardial fibers are the most sensitive to an increase in afterload, LV-LS decreases [21]. This finding is in agreement with previous results showing correlations and associations between aortic root diameters and global LV-LS [22]. The LV often attempts to compensate for this through an increased workload of the transmural/radial fibers, which explains the trend toward a higher LV-RS. In contrast, the aortic valve area (AVA) exhibited a different pattern. While the majority of healthy adults present with a larger end-systolic than end-diastolic AVA, 30% of cases paradoxically display a larger end-diastolic area. Compared to individuals with a larger end-systolic AVA, those with a larger end-diastolic area demonstrated reduced basal radial and longitudinal left ventricular (LV) strains, along with lower basal LV rotation [23]. An important question is whether similar or even more pronounced alterations occur in certain pathological conditions (in the presence of lower or larger parameters, e.g., strains, aortic dimensions), which projects the necessity of conducting further investigations [19,20]. Such as in the presence of aortic valve stenosis (AS), since it is known that global LV-LS is linked to long-term outcomes and rapid AS progression in moderate AS [24]. An additional interesting question is the clinical significance and potential prognostic value of these calculated parameters and the presence or absence of their associations in certain pathologies, like in valvular abnormalities and following their invasive management. Furthermore, although aortic stiffness parameters directly accounting for blood pressure metrics were not calculated, it is well-established that higher pulse pressure in older individuals is associated with a smaller aortic lumen area, greater aortic wall stiffness and thickness, and larger LV volume. This coexistence of a larger ventricular volume and a smaller aortic lumen in relation to higher pulse pressure suggests a mismatch in hemodynamic load accommodation between the heart and the aorta in the elderly. Future studies are warranted to investigate the specific role of LV strains in this pathophysiological interplay, both in healthy individuals and in various disease states [25].

**Limitations section.** Several potential limitations should be considered when interpreting the results of this study:

- Although the study population consisted of carefully screened, apparently healthy volunteers, the presence of underlying, subclinical pathological conditions cannot be entirely ruled out, as more exhaustive diagnostic and exclusion testing was not performed.
- The image quality and overall usability of 3DSTE remain inferior to conventional 2D echocardiography, primarily due to its lower spatial and temporal resolution, as well as hardware-related factors like footprint size and matrix-array transducer technology, which may restrict its clinical utility [7,8,9,10,11].
- The current study protocol did not incorporate validation or comparative analysis of the echocardiographic parameters against other gold-standard advanced cardiac imaging modalities, such as cardiovascular magnetic resonance or computed tomography [4,5,26,27].
- While 3DSTE is a highly versatile tool capable of comprehensive cardiac chamber quantification and detailed anatomical assessment of other cardiac chambers and valves, these specific evaluations fell outside the predefined scope of the present investigation [4,5,6,7,8,9,10,11,12].
- It would have been possible to calculate aortic stiffness parameters with a combination of using AAD parameters and blood pressure values. However, making such measurements was not within the scope of this study [13,18].
- Furthermore, a notable limitation of this study is the methodology used for patient stratification. Due to the lack of established clinical reference guidelines and predefined normal value thresholds for ascending aorta dimensions and 3DSTE-derived LV strains in the current literature, patients were divided into three groups based on the mean ± SD of the cohort. We acknowledge that this statistical approach is data-driven and introduces an inherent risk of circular reasoning, which may also result in unequal group sizes. It is also important to note that multiple subgroup analyses were conducted without adjusting for multiple comparisons, which increases the likelihood of a type I error. Consequently, these findings must be interpreted strictly as exploratory. Readers should exercise caution when evaluating these trends, as they require verification in larger, independent external cohorts before establishing any clinical conclusions.
- Our study has certain limitations regarding statistical power. The post hoc power analysis reached 0.68 for our primary endpoints, which is lower than the recommended 0.80 threshold. This lower power limits our ability to detect minor variations, particularly in regional (apical, midventricular, and basal) strain values, increasing the potential for type II (false negative) errors. Future multi-center studies with a larger cohort are warranted to confirm these regional strain dynamics and ensure optimal statistical power.
- Additionally, the interpretation of our findings warrants appropriate caution. Due to the exploratory nature of the study, the relatively small sample size, and the modest strength of the reported associations, these preliminary trends require validation in larger, multi-center cohorts.

## 5. Conclusions

In conclusion, a finely tuned interaction exists between LV deformation and ascending aortic size, even in healthy individuals free of cardiovascular pathology. Specifically, larger aortic dimensions are characterized by a distinct contraction pattern of reduced LV-LS and a trend toward higher LV-RS, regardless of the cardiac cycle phase. These findings demonstrate that altered LV mechanics show associations with larger aortic diameters even within normal physiological ranges, highlighting the significant role 3DSTE could play in this type of clinical evaluation.

## Figures and Tables

**Table 1:** Clinical and two-dimensional echocardiographic data.

Data	Measures
**Clinical** **data**	
***n***	93
**Mean age (years)**	30.6 ± 10.0
**Males (%)**	54 (58)
**Systolic blood pressure (mmHg)**	123 ± 3
**Diastolic blood pressure (mmHg)**	82 ± 1
**Heart rate (bpm)**	72 ± 2
**Weight (kg)**	72.3 ± 19.6
**Height (cm)**	172.2 ± 12.2
**Body mass index (kg/m^2^)**	24.3 ± 3.2
**Two-dimensional echocardiographic data**	
**LA diameter (mm)**	37.9 ± 2.9
**LV end-diastolic diameter (mm)**	48.6 ± 3.1
**LV end-systolic diameter (mm)**	32.6 ± 3.0
**LV end-diastolic volume (mL)**	107.6 ± 23.4
**LV end-systolic volume (mL)**	37.9 ± 8.2
**Interventricular septum (mm)**	8.9 ± 1.1
**LV posterior wall (mm)**	8.8 ± 1.1
**LV ejection fraction (%)**	65.1 ± 3.1
**Early diastolic mitral inflow velocity—E (cm/s)**	82.1 ± 16.9
**Late diastolic mitral inflow velocity—A (cm/s)**	57.6 ± 11.6

**Abbreviations:** LA = left atrial, LV = left ventricular.

**Table 2:** Ascending aortic diameters and left ventricular strains in different end-diastolic ascending aortic diameter groups.

	AoAsc-D ≤ 25.1 mm (*n* = 13)	25.1 mm < AoAsc-D < 33.3 mm (*n* = 64)	33.3 mm ≤ AoAsc-D (*n* = 16)
**AoAsc-D (mm)**	22.8 ± 1.4	28.8 ± 2.4 *	35.4 ± 1.8 *†
**AoAsc-S (mm)**	25.2 ± 1.9	31.8 ± 2.7 *	38.0 ± 1.6 *†
**basal LV-RS (%)**	25.5 ± 11.1	31.9 ± 13.0	32.9 ± 11.6
**midventricular LV-RS (%)**	25.1 ± 10.0	30.3 ± 11.3	32.4 ± 13.5
**apical LV-RS (%)**	17.4 ± 6.6	19.0 ± 9.2	19.7 ± 11.3
**global LV-RS (%)**	21.1 ± 7.2	25.8 ± 9.8	27.4 ± 11.1
**mean segmental LV-RS (%)**	23.4 ± 6.5	28.1 ± 9.2	29.4 ± 10.9
**basal LV-CS (%)**	−27.4 ± 5.8	−25.1 ± 5.0	−27.0 ± 5.5
**midventricular LV-CS (%)**	−31.3 ± 5.6	−30.0 ± 6.0	−33.0 ± 6.6
**apical LV-CS (%)**	−34.4 ± 8.1	−32.9 ± 10.1	−31.3 ± 9.0
**global LV-CS (%)**	−30.0 ± 4.9	−28.0 ± 5.0	−29.5 ± 4.8
**mean segmental LV-CS (%)**	−30.6 ± 4.7	−28.9 ± 4.8	−30.3 ± 5.0
**basal LV-LS (%)**	−20.3 ± 4.2	−21.1 ± 4.4	−18.3 ± 3.8 †
**midventricular LV-LS (%)**	−14.8 ± 4.3	−13.0 ± 3.9	−12.3 ± 3.3
**apical LV-LS (%)**	−18.8 ± 6.0	−18.2 ± 6.1	−15.5 ± 3.7
**global LV-LS (%)**	−17.0 ± 2.6	−16.6 ± 2.4	−14.4 ± 2.3 *†
**mean segmental LV-LS (%)**	−17.9 ± 3.3	−17.3 ± 2.3	−15.4 ± 2.2 *†

**Abbreviations.** AoAsc-D = end-diastolic ascending aortic diameter; AoAsc-S = end-systolic ascending aortic diameter; LV = left ventricular; RS = radial strain; CS = circumferential strain; LS = longitudinal strain. * *p* < 0.05 vs. AoAsc-D ≤ 25.1 mm; † *p* < 0.05 vs. 25.1 mm < AoAsc-D < 33.3 mm.

**Table 3:** Ascending aortic diameters and left ventricular strains in different end-systolic ascending aortic diameter groups.

	AoAsc-S ≤ 27.7 mm (*n* = 13)	27.7 mm < AoAsc-S < 36.3 mm (*n* = 61)	36.3 mm ≤ AoAsc-S (*n* = 19)
**AoAsc-D (mm)**	23.2 ± 1.7	28.7 ± 2.5 *	34.7 ± 2.1 *†
**AoAsc-S (mm)**	24.9 ± 1.4	31.5 ± 2.1 *	38.2 ± 1.4 *†
**basal LV-RS (%)**	28.9 ± 9.5	32.0 ± 13.7	31.3 ± 10.5
**midventricular LV-RS (%)**	26.6 ± 10.7	30.7 ± 11.4	30.4 ± 12.9
**apical LV-RS (%)**	17.7 ± 5.5	18.7 ± 9.5	20.1 ± 10.5
**global LV-RS (%)**	23.2 ± 6.8	26.0 ± 10.1	25.7 ± 10.7
**mean segmental LV-RS (%)**	25.2 ± 5.9	28.2 ± 9.6	28.2 ± 10.3
**basal LV-CS (%)**	−27.8 ± 5.7	−25.3 ± 5.2	−25.9 ± 5.1
**midventricular LV-CS (%)**	−31.1 ± 5.5	−30.5 ± 6.2	−31.3 ± 6.7
**apical LV-CS (%)**	−33.9 ± 7.8	−33.2 ± 10.2	−30.8 ± 9.2
**global LV-CS (%)**	−30.0 ± 4.8	−28.3 ± 5.2	−28.2 ± 4.6
**mean segmental LV-CS (%)**	−30.6 ± 4.7	−29.2 ± 5.0	−29.2 ± 4.7
**basal LV-LS (%)**	−20.0 ± 4.4	−21.0 ± 4.3	−19.2 ± 4.4
**midventricular LV-LS (%)**	−14.2 ± 5.2	−13.3 ± 3.8	−12.3 ± 3.2
**apical LV-LS (%)**	−18.5 ± 5.8	−18.2 ± 6.2	−16.0 ± 3.9
**global LV-LS (%)**	−16.6 ± 2.8	−16.7 ± 2.5	−14.8 ± 2.4 †
**mean segmental LV-LS (%)**	−17.4 ± 3.4	−17.4 ± 2.3	−15.8 ± 2.2 †

**Abbreviations.** AoAsc-D = end-diastolic ascending aortic diameter; AoAsc-S = end-systolic ascending aortic diameter; ; LV = left ventricular, RS = radial strain; CS = circumferential strain; LS = longitudinal strain. * *p* < 0.05 vs. AoAsc-S ≤ 27.7 mm; † *p* < 0.05 vs. 27.7 mm < AoAsc-S < 36.3 mm.

**Table 4:** Ascending aortic diameters and left ventricular strains in different global left ventricular radial strain groups.

	Global LV-RS ≤ 15.7% (*n* = 13)	15.7% < Global LV-RS < 35.3% (*n* = 67)	35.3% ≤ Global LV-RS (*n* = 13)
**AoAsc-D (mm)**	28.1 ± 4.6	29.6 ± 4.2	29.6 ± 2.9
**AoAsc-S (mm)**	32.0 ± 4.6	32.1 ± 4.5	32.6 ± 2.3
**basal LV-RS (%)**	17.7 ± 7.6	30.8 ± 9.7 *	48.2 ± 11.7 *†
**midventricular LV-RS (%)**	15.5 ± 3.2	28.5 ± 6.7 *	51.6 ± 8.5 *†
**apical LV-RS (%)**	11.6 ± 6.9	17.8 ± 6.7 *	31.1 ± 11.6 *†
**global LV-RS (%)**	11.6 ± 2.6	24.6 ± 5.0 *	43.5 ± 6.9 *†
**mean segmental LV-RS (%)**	15.4 ± 2.5	26.7 ± 4.9 *	45.2 ± 7.0 *†
**basal LV-CS (%)**	−22.6 ± 5.4	−25.9 ± 5.1 *	−28.1 ± 4.8 *
**midventricular LV-CS (%)**	−26.9 ± 5.8	−30.7 ± 5.5 *	−34.6 ± 7.8 *†
**apical LV-CS (%)**	−32.2 ± 10.2	−32.2 ± 9.1	−36.0 ± 11.9
**global LV-CS (%)**	−25.8 ± 4.7	−28.4 ± 4.5 *	−31.7 ± 6.3 *†
**mean segmental LV-CS (%)**	−26.6 ± 4.7	−29.3 ± 4.3 *	−32.5 ± 6.3 *†
**basal LV-LS (%)**	−18.4 ± 3.0	−20.7 ± 4.6	−20.9 ± 3.8
**midventricular LV-LS (%)**	−11.7 ± 3.2	−13.1 ± 4.0	−14.5 ± 4.1
**apical LV-LS (%)**	−19.9 ± 5.0	−17.9 ± 5.5	−15.8 ± 7.5
**global LV-LS (%)**	15.2 ± 2.3	−16.4 ± 2.6	−16.6 ± 2.9
**mean segmental LV-LS (%)**	−16.3 ± 2.2	−17.1 ± 2.6	−17.2 ± 2.8

**Abbreviations.** AoAsc-D = end-diastolic ascending aortic diameter; AoAsc-S = end-systolic ascending aortic diameter; ; LV = left ventricular, RS = radial strain; CS = circumferential strain; LS = longitudinal strain. * *p* < 0.05 vs. global LV-RS ≤ 15.7%; † *p* < 0.05 vs. 15.7% < global LV-RS < 35.3%.

**Table 5:** Ascending aortic diameters and left ventricular strains in different global left ventricular circumferential strain groups.

	Global LV-CS ≤ −23.5% (*n* = 10)	−23.5% < Global LV-CS < −33.5% (*n* = 70)	−33.5% ≤ Global LV-CS (*n* = 13)
**AoAsc-D (mm)**	28.2 ± 2.7	29.2 ± 4.1	30.0 ± 4.5
**AoAsc-S (mm)**	31.2 ± 2.5	31.9 ± 4.5	32.7 ± 4.2
**basal LV-RS (%)**	34.0 ± 14.1	30.2 ± 11.4	37.3 ± 15.9 †
**midventricular LV-RS (%)**	27.2 ± 14.3	28.6 ± 9.5	41.6 ± 15.0 *†
**apical LV-RS (%)**	11.7 ± 4.5	17.4 ± 7.2	32.2 ± 10.0 *†
**global LV-RS (%)**	23.2 ± 10.9	24.3 ± 8.1	35.3 ± 12.6 *†
**mean segmental LV-RS (%)**	25.9 ± 10.1	26.4 ± 7.6	37.6 ± 12.3 *†
**basal LV-CS (%)**	−22.2 ± 4.9	−25.1 ± 4.4 *	−32.4 ± 4.7 *†
**midventricular LV-CS (%)**	−22.8 ± 4.2	−30.1 ± 4.4 *	−40.7 ± 4.0 *†
**apical LV-CS (%)**	−22.3 ± 8.5	−32.3 ± 8.0 *	−43.2 ± 9.4 *†
**global LV-CS (%)**	−21.1 ± 2.8	−27.9 ± 3.1 *	−37.5 ± 2.6 *†
**mean segmental LV-CS (%)**	−22.4 ± 2.9	−28.8 ± 3.0 *	−38.2 ± 2.6 *†
**basal LV-LS (%)**	−21.4 ± 3.9	−20.6 ± 4.5	−19.4 ± 3.7
**midventricular LV-LS (%)**	−13.0 ± 3.4	−12.6 ± 3.7	−16.3 ± 4.1 †
**apical LV-LS (%)**	−16.2 ± 5.5	−17.7 ± 5.6	−19.4 ± 6.9
**global LV-LS (%)**	−16.2 ± 2.8	−16.1 ± 2.5	−17.4 ± 2.6 †
**mean segmental LV-LS (%)**	−16.9 ± 2.6	−16.9 ± 2.5	−18.3 ± 2.3 †

**Abbreviations.** AoAsc-D = end-diastolic ascending aortic diameter; AoAsc-S = end-systolic ascending aortic diameter; LV = left ventricular, RS = radial strain; CS = circumferential strain; LS = longitudinal strain. * *p* < 0.05 vs. global LV-CS ≤ -23.5%; † *p* < 0.05 vs. -23.5% < global LV-CS < -33.5%.

**Table 6:** Ascending aortic diameters and left ventricular strains in different global left ventricular longitudinal strain groups.

	Global LV-LS ≤ −13.7% (*n* = 15)	−13.7% < Global LV-LS < −18.9% (*n* = 61)	−18.9% ≤ Global LV-LS (*n* = 17)
**AoAsc-D (mm)**	31.6 ± 4.4	28.7 ± 4.1 *	28.8 ± 2.9 *
**AoAsc-S (mm)**	34.0 ± 4.6	31.5 ± 4.3 *	31.8 ± 3.3
**basal LV-RS (%)**	30.5 ± 13.0	31.8 ± 12.4	30.9 ± 12.9
**midventricular LV-RS (%)**	28.8 ± 13.8	30.0 ± 10.8	31.3 ± 12.5
**apical LV-RS (%)**	19.4 ± 11.7	17.8 ± 8.5	21.9 ± 9.2
**global LV-RS (%)**	24.7 ± 12.1	25.4 ± 9.2	26.8 ± 9.6
**mean segmental LV-RS (%)**	27.1 ± 11.8	27.6 ± 8.5	28.8 ± 9.8
**basal LV-CS (%)**	−25.1 ± 4.4	−26.0 ± 5.2	−25.4 ± 6.2
**midventricular LV-CS (%)**	−30.4 ± 6.3	−30.3 ± 5.6	−32.7 ± 7.8
**apical LV-CS (%)**	−30.8 ± 10.7	−31.7 ± 8.9	−38.3 ± 9.8 *†
**global LV-CS (%)**	−27.5 ± 5.1	−28.2 ± 4.4	−30.6 ± 6.3
**mean segmental LV-CS (%)**	−28.5 ± 4.9	−29.1 ± 4.3	−31.4 ± 6.3
**basal LV-LS (%)**	−16.6 ± 3.2	−20.9 ± 4.1 *	−22.5 ± 4.2 *
**midventricular LV-LS (%)**	9.9 ± 2.5	−12.7 ± 3.2 *	−17.8 ± 3.6 *†
**apical LV-LS (%)**	−14.9 ± 4.0	−17.1 ± 5.1	−22.9 ± 6.6 *†
**global LV-LS (%)**	−12.6 ± 1.5	−16.1 ± 1.4 *	−20.2 ± 1.0 *†
**mean segmental LV-LS (%)**	−13.6 ± 1.3	−16.9 ± 1.4 *	−20.8 ± 1.6 *†

**Abbreviations.** AoAsc-D = end-diastolic ascending aortic diameter; AoAsc-S = end-systolic ascending aortic diameter; ; LV = left ventricular, RS = radial strain; CS = circumferential strain; LS = longitudinal strain. * *p* < 0.05 vs. global LV-LS ≤ −13.7%; † *p* < 0.05 vs. −13.7% < global LV-LS < −18.9%.

## Data Availability

Data available on request due to restrictions, e.g., privacy or ethical restrictions.
